# A record of vapour pressure deficit preserved in wood and soil across biomes

**DOI:** 10.1038/s41598-020-80006-9

**Published:** 2021-01-12

**Authors:** Adrian Broz, Gregory J. Retallack, Toby M. Maxwell, Lucas C. R. Silva

**Affiliations:** 1 Department of Earth Sciences, University of Oregon, Eugene, 97403 USA; 2 Department of Biological Sciences, Boise State University, Boise, ID 83725 USA; 3 Environmental Studies Program, Department of Geography, University of Oregon, Eugene, 97403 USA; 4 Institute of Ecology and Evolution, University of Oregon, Eugene, 97403 USA

**Keywords:** Geochemistry, Palaeoecology, Drought, Projection and prediction, Palaeoclimate

## Abstract

The drying power of air, or vapour pressure deficit (VPD), is an important measurement of potential plant stress and productivity. Estimates of VPD values of the past are integral for understanding the link between rising modern atmospheric carbon dioxide (pCO_2_) and global water balance. A geological record of VPD is needed for paleoclimate studies of past greenhouse spikes which attempt to constrain future climate, but at present there are few quantitative atmospheric moisture proxies that can be applied to fossil material. Here we show that VPD leaves a permanent record in the slope (*S*) of least-squares regressions between stable isotope ratios of carbon and oxygen (^13^C and ^18^O) found in cellulose and pedogenic carbonate. Using previously published data collected across four continents we show that *S* can be used to reconstruct VPD within and across biomes. As one application, we used *S* to estimate VPD of 0.46 kPa ± 0.26 kPa for cellulose preserved tens of millions of years ago—in the Eocene (45 Ma) *Metasequoia* from Axel Heiberg Island, Canada—and 0.82 kPa ± 0.52 kPa—in the Oligocene (26 Ma) for pedogenic carbonate from Oregon, USA—both of which are consistent with existing records at those locations. Finally, we discuss mechanisms that contribute to the positive correlation observed between VPD and *S*, which could help reconstruct past climatic conditions and constrain future alterations of global carbon and water cycles resulting from modern climate change.

## Introduction

Vapour pressure deficit, or VPD, is the difference between the amount of moisture in the air and how much moisture the air can hold when it is saturated, with the latter depending on ambient temperature^1,2^. Changes in VPD reflect the potential for the atmosphere to extract water from terrestrial ecosystems. VPD is often monitored as a proxy for plant water stress because it is a principal control on stomatal water loss and photosynthetic carbon fixation^2^. VPD is not a meteorological parameter for climate studies because it is a relative metric of stress that varies among plant species, as inferred from their leaf functional traits, and from interactions between roots, soils, and microorganisms in the rhizosophere, which together govern responses to climate at local to global scales^3–5^. However, VPD does reflect the effect of temperature and precipitation on relative humidity^6^ and transpiration demand, which stimulates stomatal closure to minimize water loss, and thus the flow of water and nutrients from the soil through plants and ultimately to the atmosphere^7,8^. Stomatal closure in turn affects carbon (C) isotopes of plant cellulose^9–11^, which decays into soil organic matter and respired CO_2_, and as a result the ratio of stable carbon isotopes (δ^13^C) can be passed on to pedogenic carbonates^9^. Oxygen (O) isotopes are also impacted by VPD, responding as a function of stomatal closure as well as independently of transpiration demands, and stable oxygen isotope ratios (δ^18^O) can be used to isolate VPD-imposed stress from other environmental factors that control δ^13^C of cellulose^12,13^. Numerous processes are known to affect the fractionation of C and O isotopes in plants and soils. Here, we explore the mechanisms that relate VPD with changes in δ^13^C and δ^18^O measured in cellulose and pedogenic carbonates and make a case for using those ratios as a proxy for climatic conditions.

### Stable isotope ratios of plant cellulose in response to drought

**Figure 1:**
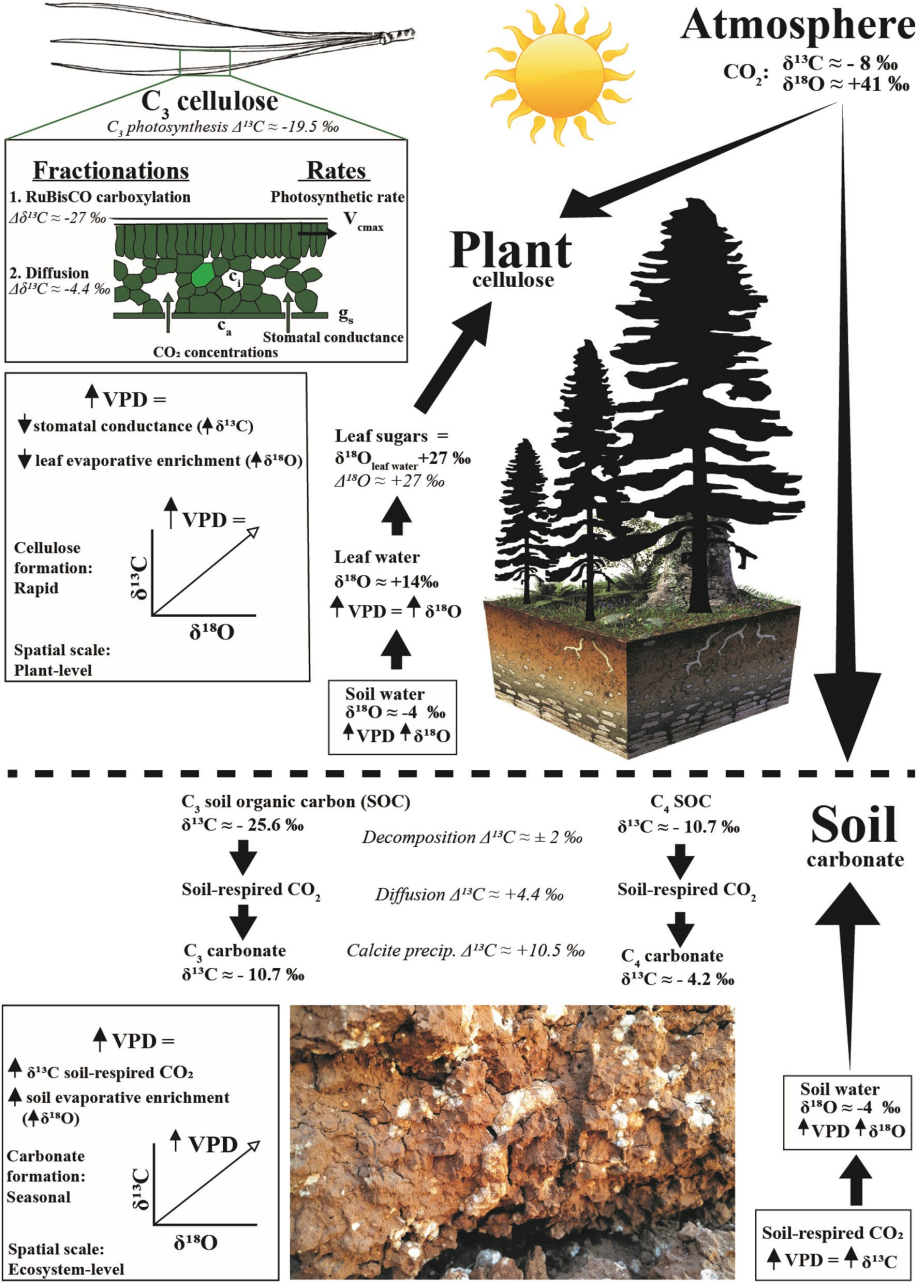
Diagram of typical stable carbon and oxygen isotope values measured in cellulose (top panel) and soil carbonate (bottom panel). Fractionation steps (listed in italics) and the influence of vapour pressure deficit (VPD) for cellulose and pedogenic carbonates use a modern value for δ^13^C of atmospheric CO_2_. Stable isotope values for C_3_ plant cellulose^20,21^ and soil carbonate^22^ represent rough approximations and are expected to vary significantly with differences in geographic location, environmental conditions and concentration of atmospheric CO_2._ Values are on the Vienna Standard Mean Ocean Water (SMOW) and Vienna Pee Dee Belemnite (PDB) scales for δ^18^O and δ^13^C values, respectively.

Cellulose δ^13^C values in C_3_ plants reflect the ratio of intercellular (c_i_) to atmospheric (c_a_) partial pressure of CO_2_ and CO_2_ fixation by RuBisCo, which yield δ^13^C fractionations of − 4.4‰ and − 27‰, respectively^14^ (Fig. 1). Physiological stress alters the cellulose δ^13^C value via its effects on stomatal conductance and the internal concentration of CO_2_ in leaves^10^. On the other hand, cellulose δ^18^O values reflect the isotopic ratio of the source water^15^ which depends on condensation temperature and Rayleigh distillation processes^16^. Leaf water oxygen enrichment is dependent in part on the ratio of intercellular to atmospheric vapour pressures (e_i_ and e_a,_ respectively) while the ratio of c_i_ and c_a_ is approximately related to δ^13^C values recorded in cellulose and other compounds. Decreased stomatal conductance combined with evaporative enrichment of leaf water ^18^O causes both ^13^C and ^18^O to increase simultaneously in cellulose (Fig. 1), which can lead to a positive correlation between δ^13^C and δ^18^O when water stress exerts a significant physiological limitation on plant-to-air C and O exchange^12,17^. Thus, in areas where elevated VPD limits plant growth, C and O isotope ratios show positive covariance in plant cellulose, and the slope of the relationship is related to VPD^8,17–19^.

**Figure 2:**
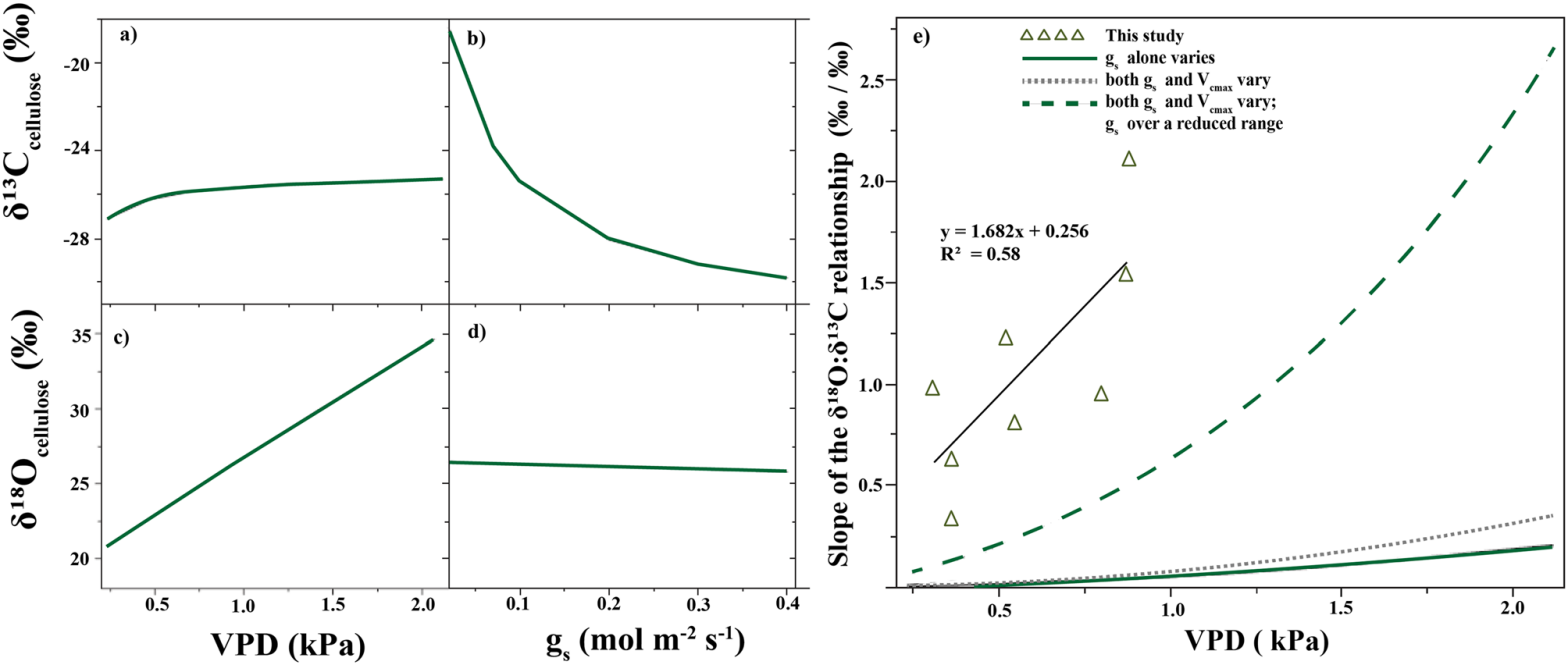
Summary output of mechanistic models developed to describe a causal relationship between VPD and δ^18^O:δ^13^C slopes in tree-ring cellulose (**a**–**d**) and in relation to our own observations of modern trees across biomes (**e**). Modeled δ^18^O and δ^13^C ratios are calibrated for *Pinus radiata* trees when: (**a**) and (**c**) vapour pressure deficit (VPD) varies; (**b**) and (**d**) stomatal conductance (*g*_s_) varies, under constant source water (δ^18^O at − 8.0‰; model adapted from Barbour et al.^8^). Air temperature was kept constant at 20 °C, and stomatal conductance (*g*_s_) varied between 0.02 and 0.48 mol m^−2^ s^−1^. Photosynthetic capacity (*V*_cmax_) at the given temperature varied between 24 and 34 mmol m^−2^ s^−1^; *V*_cmax_ variation alone showed little influence on δ^18^O and δ^13^C^8^. Model defaults were vapour pressure deficit = 0·94 kPa, *g*_s_ = 0.19 mol m^−2^ s^−1^ (on a projected leaf area basis) and *V*_cmax_ = 30 mmol m^−2^ s^−1^ and constant source water δ^18^O of – 8.0‰. (**e**) Shows the modeled relationships between vapour pressure deficit (VPD) and the change in slope of the δ^18^O and *δ*^*13*^*C* relationship when variation in δ^13^C is driven by changes in *g*_s_ alone, or by large changes in both *g*_s_ and *V*_cmax_, or by small variation in *g*_s_ and large variation in *V*_cmax_. We plotted our compiled global observations (triangles) of cellulose δ^18^O:δ^13^C slopes and annual average VPD in contemporary needle-bearing taxa from around the world which showed a positive and significant relationship between δ^18^O and δ^13^C (Table S5). For ease of comparison with the Barbour et al. model^8^, here we plotted our modern cellulose data to show δ^18^O:δ^13^C slopes (*S*^-1^), whereas δ^13^C:δ^18^O slopes (*S*) are used as previously suggested for paleo-VPD estimates (see Methods for details).

Previous studies have identified possible mechanisms by which δ^13^C:δ^18^O slope varies with VPD in tree–ring cellulose^8,12,17^. For example, the data-enabled model proposed by Saurer et al.^17^ indicates that slope is a function of the ratio of e_i_/e_a_ and c_i_/c_a_, which varies across species and with relative humidity. That model was tested using three tree genera (*Picea, Fagus and Pinus* sp*.*) at sites with markedly different soil moisture indices. The difference in slope values between species indicated a stronger dependence on c_i_/c_a_ which suggests a species-dependent relationship between slope and VPD, e.g., *Fagus* reacted more strongly in terms of stomatal downregulation of gas exchange to moisture conditions than did *Picea*^12,17^*.* Consistent with Saurer et al., a mechanistic model proposed by Barbour et al. can be used to relate slope of the δ^18^O:δ^13^C relationship to annual VPD in cellulose of *Pinus* trees under varying stomatal conductance (*g*_s_) and photosynthetic capacity (*V*_cmax_)^8^. Here, we summarize the main outputs of that model (Fig. 2) to illustrate how the slope of the δ^18^O and δ^13^C relationship increases with increasing VPD if *g*_s_ varies alone, or in tandem with *V*_cmax_. This model was originally tested with *Pinus radiata* from three sites in New Zealand which all showed positive and significant correlation between δ^18^O and δ^13^C ^8^, and notably the slope of the relationship (0.30‰ change in δ^18^O per 1‰ change in δ^13^C) is identical to the slope found in *P. sylvestris* by Saurer et al.^17^ which support the hypothesis that δ^13^C:δ^18^O slope can be used to infer VPD-induced stress for different species of conifers.

Scheidegger et al.^12^ predict with a conceptual model the occurrence of negative slope between δ^13^C and δ^18^O when c_i_, g_s_ and δ^18^O increase (while δ^13^C decreases and V_cmax_ is held constant), or when V_cmax_ decreases and g_s_ is held constant. On the other hand, drought-induced changes in stomatal conductance (high VPD) increase both δ^13^C (via stomatal conductance) and δ^18^O (via changes to c_a_/c_i_), so positive correlations between δ^13^C and δ^18^O are expected for time-series data from modern tree-ring cellulose^23^. Moreover, a positive linear relationship implies that c_i_/c_a_ depends linearly on e_i_/e_a_ and is influenced by changes in VPD^17^. Indeed, experimental studies have shown that c_i_/c_a_ decreases linearly with increased VPD in C_3_ plants^24^ which supports the hypothesis that VPD controls the slope value of δ^13^C and δ^18^O during periods of water stress when other factors are held constant. Building on those findings, we examined the relationship between δ^18^O and δ^13^C slope and annual average VPD, and then compared our data compilation to the modelled response to VPD flux (Fig. 2e). If we fit a linear regression to modelled slope versus VPD reported by Barbour et al.^8^, we get slopes of 0.11 when g_s_ alone varies, 0.17 when both g_s_ and V_cmax_ vary, and 1.34 when *g*_s_ and *V*_cmax_ vary while *g*_s_ varies over a limited range (Table S6). Our empirically determined slope from cellulose around the world is 1.68, which is consistent with the highest modelled slope for *P. radiata* (1.34) when both *g*_s_ and *V*_cmax_ varies while *g*_s_ varies over a limited range (Fig. 2e, Table S6). Our compiled global dataset for cellulose includes three genera of pine (*P. ponderosa, P. sylvestris, P. radiata*) as well as *Larix sibirica* and *Tsuga canadensis *(Table S5, which could explain the difference in absolute value (y-intercept) between our data and the Barbour et al. model^8^. Another factor that might have contributed to those differences is the variations in source water δ^18^O across sites, which can affect the y-intercept irrespectively of potential differences in species-specific traits. Despite those differences, our data show a remarkably consistent slope relative to the cellulose model, which points to the possibility of new applications across species and spatial scales. Together, mechanistic models and global observations suggest that VPD-induced stress can be inferred from correlations between δ^13^C and δ^18^O values. However, functional traits across species and/or genera modulate differences in δ^13^C and δ^18^O excursions in response to drought^25^ and thus differences in *S* between species are expected with increasing VPD. As such, any use of a VPD proxy should only be applied to fossil wood where identification to the genus level is possible.

### Stable isotope ratios in pedogenic carbonate in response to drought

Changes in VPD are expected to cause changes in δ^13^C and δ^18^O values in pedogenic carbonate, but the mechanisms leading to those correlations are different than in cellulose. Pedogenic carbonate (calcite, CaCO_3_) forms in soil where potential evaporation exceeds evapotranspiration, most often in arid to subhumid regions which receive less than ~ 100 cm of precipitation annually^26^. The sources of C in pedogenic carbonate are from autotrophic root-respired CO_2_, heterotrophic decomposition of organic matter by soil microbes and from the diffusion of atmospheric CO_2_ into the soil matrix^26–28^ (Fig. 1). Soil-respired CO_2_ is often an order of magnitude greater in concentration than atmospheric CO_2_ which creates a diffusion gradient that drives net flow of CO_2_ to the atmosphere^27^. Therefore, the carbon isotopic composition of pedogenic carbonate is most sensitive to the isotopic composition of soil-respired CO_2_^11^. Other variables that control the δ^13^C of soil carbonate are (1) the proportion of C_3_–C_4_ plants growing at the site; (2) root and microbial respiration rates, which are sensitive to changes in VPD; and (3) the CO_2_ concentration of the atmosphere^11,29^. The carbon isotopic signature of water stress in C_3_ plants is passed on to soil-respired CO_2_ because the original δ^13^C isotope composition of the plant community is preserved (± 2‰) in soil-respired CO_2_ generated during aerobic decay of soil organic matter^9,11,30^. Soil-respired CO_2_ then equilibrates with soil water to form pedogenic carbonate during seasonal drying of the soil^9,27,31^.

The source of O in pedogenic carbonates is from meteoric water, which infiltrates into the soil matrix and becomes soil water^32^. Pedogenic carbonate is assumed to be in O isotopic equilibrium with soil water and thus carbonate δ^18^O values are used to constrain paleotemperature and/or paleoelevation^33,34^. Oxygen isotope ratios of pedogenic carbonate do not carry a plant signal because plant compounds show little O isotopic exchange with soil water during decomposition^35^. Therefore, the decay of cellulose into soil organic matter and respired CO_2_ is expected to pass the carbon isotopic signature of the plant community to pedogenic carbonates (Fig. 1), which can also record changes in moisture regime when the effect of evaporative enrichment on δ^18^O of plant and soil water is considered^36–38^.

In the following sections, we show that cellulose and carbonate δ^13^C:δ^18^O slopes (*S*) are strong predictors of VPD, such that *S* may be used to infer climatic conditions at spatiotemporal scales that go beyond those of tree-ring studies. Given that profound changes in atmospheric moisture are predicted with climate change^7^, VPD records would be useful for inferring past climate conditions and reducing uncertainties in future climate projections. We posit that atmospheric VPD is preserved in isotope ratios of soil carbonate, just as in cellulose, such that suitably preserved fossil wood and paleosol carbonate can be used as a proxy for VPD of past environments. As proof of concept, we use previously published data to develop *S*-to-VPD transfer functions using fossil cellulose from Arctic *Metasequoia* during the Eocene^39^ and pedogenic carbonate formed during the Oligocene in calcareous paleosols from Oregon^40^, both of which show a positive correlation between δ^13^C and δ^18^O similar to those found for modern cellulose and carbonate samples.

## Results

**Figure 3:**
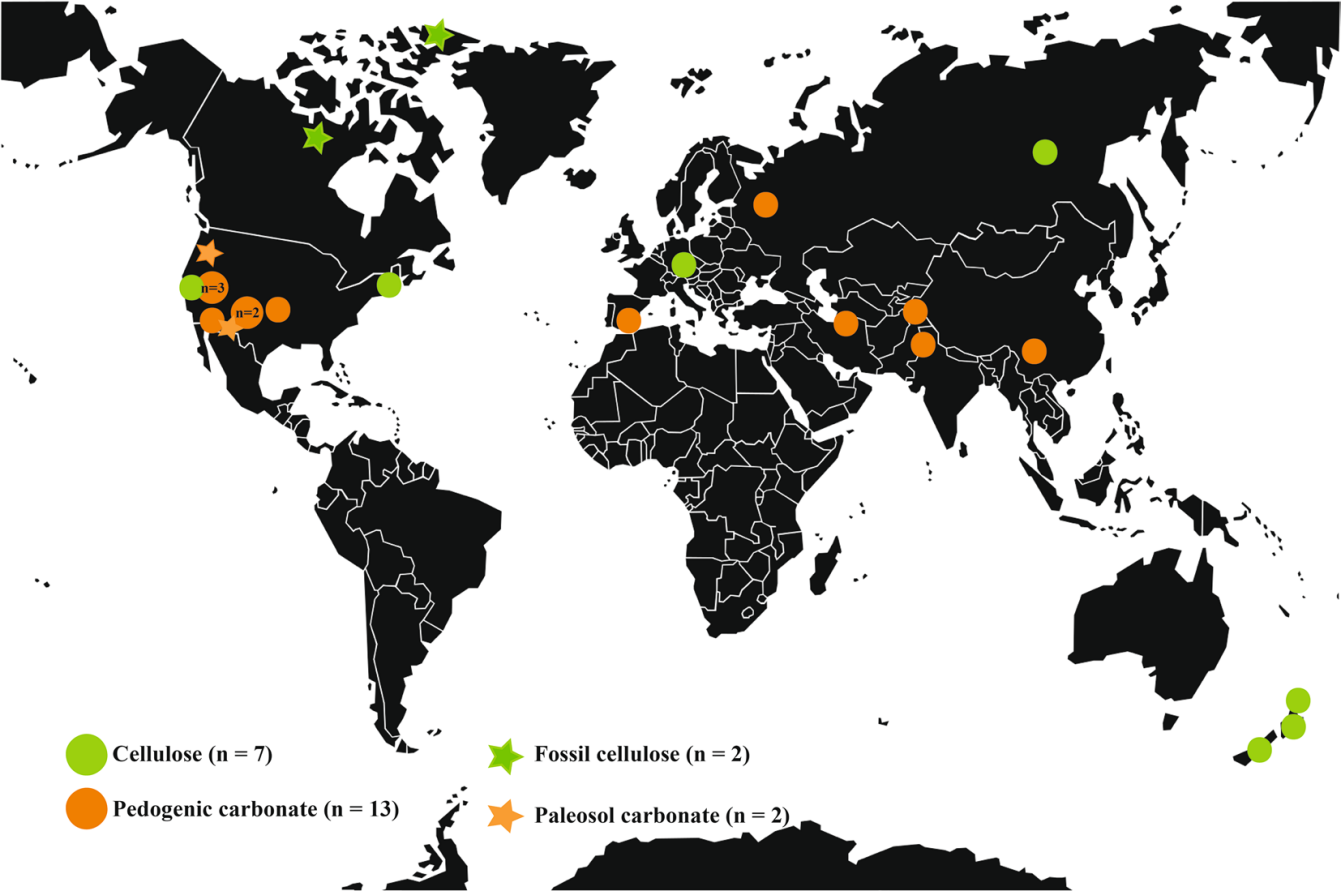
Approximate locations where modern cellulose (green circles, n = 8) and pedogenic carbonate (orange circles, n = 13) stable isotope data were collected (see Table S5). The locations of fossil cellulose (green stars, n = 2) and pedogenic carbonate (orange stars, n = 2) used for paleo-VPD estimates are also noted.

**Figure 4:**
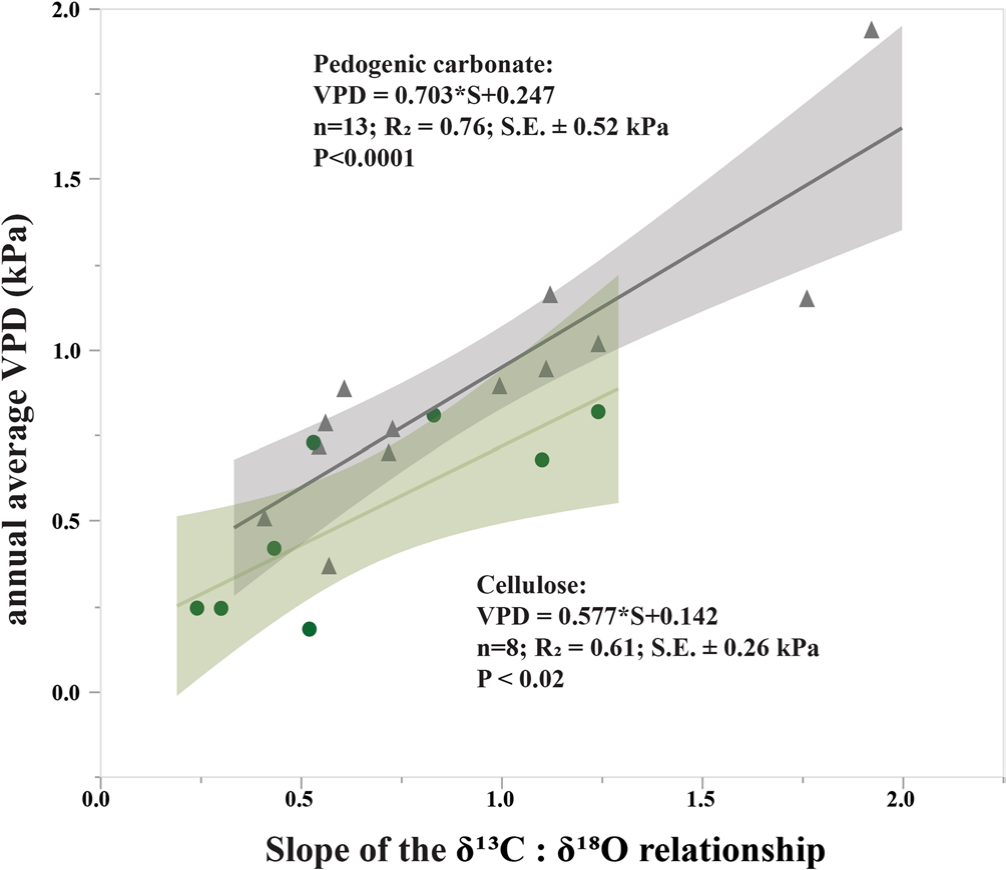
Least-squares regressions for modern samples from around the world relating vapour pressure deficit (VPD) and the slope of the positive correlation between δ^13^C and δ^18^O (*S*) in modern tree-ring cellulose (circles) and modern pedogenic carbonate (triangles). No fossil data are shown in this figure. Shaded areas are 95% confidence prediction intervals. Propagated error (S.E.) for VPD predictions using cellulose (± 0.26 kPa) and pedogenic carbonate (± 0.52 kPa) were calculated from A) the standard error of each modern data point’s δ^13^C:δ^18^O slope when slope was calculated from raw data; B) the standard error of modern VPD measurements when calculated from average climate statistics (± 0.13 kPa)^41,42^; and C) the standard error of the transfer functions.

Our contribution to the record of atmospheric VPD preserved in plants and pedogenic carbonate is a global compilation of data on stable isotopic composition of cellulose and pedogenic carbonate (Fig. 3, Supplementary data). The criteria used for the data selection (for cellulose) were: a positive and significant (*P* < 0.05) correlation between δ^13^C and δ^18^O measured in α-cellulose isolated from individual trees (needle-bearing taxa only) from 1950-present which had n > 8 data points and met model assumptions for simple linear regression. For carbonate we considered datasets reporting positive and significant correlation between δ^13^C and δ^18^O in nodular calcite gathered from individual soil profiles which also had n > 8 data points and met assumptions for simple linear regression (see Methods for details). A positive correlation between δ^13^C and δ^18^O was found when annual average VPD (VPD_annual_) exceeded ~ 0.3 kPa. Non-significant (*P* > 0.05) and/or negative correlations of δ^13^C and δ^18^O were noted in cases where VPD_annual_ was less than ~ 0.3 kPa and/or when original authors noted that drought stress was not a significant factor influencing isotope ratios (e.g., when isotopic excursions were attributed to variation in sunlight or temperature). The slope of the δ^13^C:δ^18^O relationship *(S)* in both modern plant cellulose (*S*_*c*_) and pedogenic carbonate (*S*_*k*_) is correlated with VPD_annual_ of the contemporary atmospheric systems (Fig. 4). In other words, differences in *S* between dry and wet ecosystems appear to have been preserved over time, even though significant climatic variability can occur within each system. The coefficient of determination of the correlation between *S*_*c*_ and VPD_annual_ according to Eq. (1) is r^2^ = 0.61 (n = 8; s.e. ± 0.26 kPa; *P* < 0.02). The coefficient of determination of the correlation between *S*_*k*_ and VPD_annual_ according to Eq. (2) is r^2^ = 0.76 (n = 13; s.e. =  ± 0.52 kPa; *P* < 0.0001). Additionally, a negative correlation between *S*_*k*_ and annual relative humidity was observed (Fig. S1).

1 $$VPD = 0.577\cdot {S}_{c} + 0.142$$

2 $$VPD = 0.703\cdot {S}_{k} + 0.247$$

**Figure 5:**
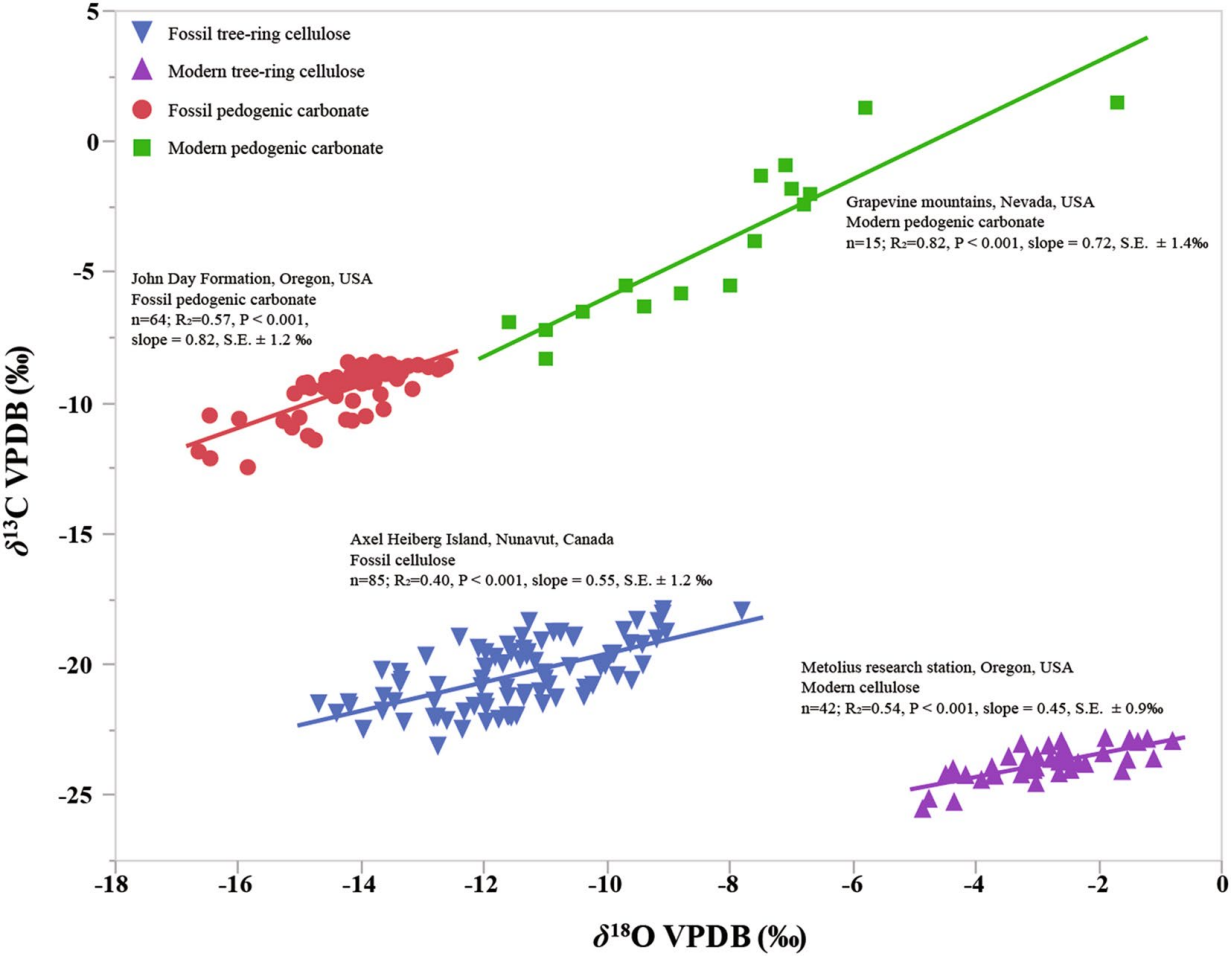
Relationship between δ^13^C and δ^18^O in select modern and fossil samples (from Fig. 3) used for estimating paleo-VPD. Fossil samples (circles and triangles) of cellulose and carbonate are from the Eocene (45 Ma) and Oligocene (26 Ma), respectively. All cellulose δ^18^O values were recalibrated to the VPDB scale (Table S7). Both modern and fossil isotopic datasets are listed in Table S3 (for cellulose) and Table S4 (for carbonate).

The transfer functions generated from modern tree-ring cellulose isotope training datasets (Eqs. 1 and 2) can be used to estimate VPD_annual_ during the Eocene and Oligocene and are compared with other independent records as proof-of-concept. The *S*_*c*_ in Eocene (45 Ma) tree-ring cellulose of *Metasequoia* from Axel Heiberg Island, Nunavut, Canada^9,10^ was 0.55 (n = 85; r^2^ = 0.40, *P* < 0.001, s.e. ± 1.2 ‰). This gives paleo-VPD values of 0.46 kPa ± 0.26 kPa (Fig. 5). Eocene relative humidity of 67% from δH:δ^18^O slope and a MAT estimate of 13.2 ± 2 °C^43^ allows back calculation using Eq. (3) (see Methods) to a predicted VPD_annual_ of 0.49 kPa, which is remarkably consistent with our new paleo-VPD estimate derived from *S*_*c*_. An additional estimate of Eocene VPD from mummified tree-ring cellulose of early Eocene (53.5 Ma) *Piceoxylon* from Lac De Gras, Canada (Table S3) show *S*_*c*_ of 0.32 (n = 84; r^2^ = 0.13, *P* < 0.007, s.e. ± 0.80‰), which indicates a paleo-VPD estimate of 0.30 kPa ± 0.26 kPa. It should be noted that the original authors concluded that if the first 8 “juvenile” tree rings are excluded from the analysis the remaining samples are not significantly correlated, so caution is necessary in interpreting this dataset. Nevertheless, as a second test of the VPD proxy the *Piceoxylon* dataset, which included juvenile rings predicted VPD of 0.30 kPa ± 0.26 kPa, which is consistent with the mean annual temperature estimate of 11.4 °C ± 1.8 °C derived from transfer functions and modeled RH values ranging from 64 to 83%^23^ for early Eocene polar forests. The propagated error for the transfer function is nearly as large as the estimate for VPD and the correlation coefficient is low so, here too, cautious interpretation is necessary, but assuming atmospheric CO_2_ of 915 ppmv^44^ and low VPD across Arctic Canada during that period, we conclude that our record captured the early Eocene “hothouse” climate described in previous studies.

Late Oligocene (26 Ma) pedogenic carbonate from the Turtle Cove Member of the John Day Formation in central Oregon^40^ had *S*_*k*_ of 0.82 (n = 64; r^2^ = 0.57, *P* < 0.001, s.e. ± 1.2‰). This gives a VPD of 0.82 kPa ± 0.52 kPa for late Oligocene (26 Ma) calcareous paleosols of the Turtle Cove Member of the John Day Formation in central Oregon (Fig. 4), consistent with mineralogical and paleobotanical evidence for dramatic stepwise cooling and drying through the Eocene–Oligocene boundary^45,46^. An additional estimate of early Eocene (~ 55 Ma) VPD was derived from pedogenic carbonate from the Hannold Hill Member of the Tornillo Formation, Big Bend National Park, Texas, USA^47^. The *S*_*k*_ was 0.444 (n = 44; r^2^ = 0.81, *P* < 0.001, s.e. ± 0.29‰) which allowed for a paleo-VPD estimate of 0.56 kPa ± 0.52 kPa. Propagated error (± 0.52 kPa) was nearly as large as the VPD estimate of this early Eocene sample so caution with this estimate is also necessary, but a lower *S*_*k*_ value in this sample compared to the Oligocene example discussed above supports the hypothesis that early Eocene VPD was less than Early Oligocene VPD. The Tornillo Formation is assumed to be to be North America’s most southerly exposure of early Paleogene continental deposits and as such cannot be directly compared to Oligocene VPD because of differences in latitude and age. However, the predicted VPD of the Tornillo Formation sample is an estimate consistent with the conclusion of the previous study^47^ about a decrease in humidity, precipitation and temperature after the Paleocene-Eocene Thermal Maximum (PETM), which is thought to have decreased the production of kaolinite and increased the accumulation of calcite in these paleosols compared to the older underlying kaolinite-rich PETM paleosols.

## Discussion

Using a compilation of isotopic data gathered from modern cellulose and carbonate samples we found a persistent record of vapour pressure deficit (VPD) preserved in plants and soils across four continents. A remarkably consistent shift in δ^13^C and δ^18^O regression slopes (*S*) occur in response to increasing aridity, assessed as increasing VPD, in both fossil plant samples and in paleosols that were buried tens of millions of years ago. Positive correlations between δ^13^C and δ^18^O ratios found in cellulose samples, which reflect species or genus-level responses to VPD, are similar to those found for pedogenic carbonate samples, which reflect ecosystem-scale responses to VPD. Taken together, our compiled data indicate that changes in *S* identified for both modern and paleo samples are directly related to VPD, and thus *S* may be used to constrain climatic conditions at spatiotemporal scales that go beyond those of tree-ring studies.

What causes slope of the δ^13^C and δ^18^O relationship to vary with atmospheric moisture deficit in pedogenic carbonate? Laboratory studies of pedogenic calcite precipitation under variable temperature and relative humidity conditions show that δ^13^C and δ^18^O slope is steeper under both higher temperature and low relative humidity during elevated CO_2_ concentrations^38^. Soil temperature, relative humidity, soil CO_2_ concentration and the saturation state of evaporating fluids (with respect to CaCO_3_) are factors that determine trends in the positive linear correlation of δ^13^C and δ^18^O in pedogenic carbonate^38^. In this way, slope steepness is increased with high evaporation rates and reduced with lower evaporation rates (Fig. S1). Since VPD is a function of RH and temperature, a plausible hypothesis is that slope steepness of δ^13^C and δ^18^O in pedogenic carbonate increases with VPD due to a proportionally greater increase in δ^13^C (relative to δ^18^O) caused by the combined effect of physiological fractionation and root contribution to the soil carbon pool. However, it should be noted that slope is strongly dependent on the timing of calcite precipitation during fluid evaporation (e.g., the saturation state of the evaporating liquids), and the steepest slopes in laboratory-precipitated calcite are from samples with the greatest soil CO_2_ concentrations and evaporation rates^38^ both of which are highly variable in the vadose zone during pedogenic carbonate formation. Despite these uncertainties, a recent dual-isotope mechanistic model of natural pedogenic carbonates show that specific covariance of δ^13^C and δ^18^O can result from a shared climatic driver like VPD, which is responsible for the change in both isotope systems^48^. Here, we find further evidence of that relationship with nearly identical slopes found for modern cellulose and carbonate data (Fig. 4).

Mixtures of C_3_ and C_4_ vegetation do not confound the relationship in the analysis of wood samples, because the cellulose of wood is created solely by the C_3_ pathway^12^. However, when C_3_ and C_4_ pathways are mixed in savanna ecosystems, the resulting effect is a major shift in carbon isotope ratios in soil organic matter^49,50^ which would alter the *S*-to-VPD relationship in pedogenic carbonate^51^, but not in cellulose samples. Although commonly observed in association with climate-induced transitions between tropical forests and savannas, that type of isotopic excursion does not affect our interpretation because our data compilation did not include tropical systems (Fig. 3). However, several sites did include mixed C_3_/C_4_ plant communities (Table S5), which do not confound the relationship with VPD but instead appear to increase the variance in δ^13^C values (Table S1). Additional variance in isotopic composition of organic matter and soil air also comes from seasonal variation in rainfall and productivity^9^, from different plant parts such as wood versus leaves^52^ and their distinct molecular composition, and differential decay of organic matter in soils^53^. The compiled dataset presented here show variance of δ^13^C up to 14.5‰ in soils receiving mixtures of C_3_ and C_4_ organic matter, and variance of δ^18^O up to 14.4‰ caused by seasonality in water inputs (Table S1).

Alteration after burial may compromise application of these transfer functions to fossils. For example, δ^18^O of pedogenic carbonate can be changed during diagenetic dewatering and recrystallization^29,54^. For silica permineralized wood, cellulose may be extracted from the silica whose δ^18^O values reflect either hydrothermal or groundwater permineralization rather than cellulose biosynthesis^55^. The application here was to unmineralized wood compressions^39,43^, and micritic pedogenic carbonate from nodules without evidence of burial recrystallization^45^. We stress that application of this VPD proxy should be exclusively to cellulose of needle-bearing taxa showing cellular permineralization with cell wall ultrastructure preservation and without replacive recrystallization. Likewise, only paleosol carbonate samples with classic pedogenic carbonate micromorphology (displacive and replacive micrite without sparry recrystallization) should be considered. Careful sampling of paleosol carbonate with micrite concentrations of 70% or greater can ensure measurement of primary and not diagenetic δ^18^O values^56^.

The relationship between VPD and plant δ^13^C:δ^18^O ratios is complex and many processes are at play, most notably photosynthetic capacity, stomatal conductance, RuBisCO fractionation, and the amount, type and timing of water inputs, all of which have been shown to alter δ^13^C values or δ^18^O values or both^3,8,12,57^. We therefore do not expect that a single mechanism would adequately explain the consistent increase in the isotope ratios in plant molecules and pedogenic carbonates. As predicted by theory, plants and soils respond differently to VPD (Fig. 1) so difference in δ^13^C:δ^18^O slope between cellulose and soil carbonate is not surprising. The plant-derived carbon input to soil integrates the effect of all coexisting species of trees, shrubs, grasses, forbs, and microorganisms in addition to vast amounts of inorganic carbon^22^. Further, there is no stomatal control on soil evaporative enrichment of oxygen. The differences in slope of modern samples can be explained by these differences in biosynthetic versus physical fractionations discussed above and based on ecological processes that drive changes in the relative contributions of multiple sources of organic carbon.

Regional studies reveal that cellulose δ^18^O values reflect the isotopic ratio of source water^15^, which at large scales depends on condensation temperature and Rayleigh distillation processes^16^. At the local scale, δ^18^O of soil water is determined by the source and amount of water inputs and by soil–plant interactions that impact soil water uptake with increasing depth^58^. Regional variations in δ^13^C of organic matter and pedogenic carbonate can be related to atmospheric CO_2_ levels, vegetation types and climatic gradients^11,59^. These regional factors explain where each of our site-specific and species-specific datasets are placed on δ^13^C and δ^18^O axes (Fig. 5), but do not explain the significant correlation of δ^13^C and δ^18^O within that site. Changes in stomatal conductance due to physiological stress can result in a spread of up to 10‰ in cellulose δ^13^C and δ^18^O^57,60^, and our dataset spans most of that range for δ^13^C (9.3‰, Table S1) but indicates a much larger range for δ^18^O (17‰), which is to be expected given the large variation in source water across time and space. Biochemical oxygen isotope fractionation during cellulose synthesis can vary between 26‰ and 31‰ depending on temperature and VPD^61^, but another potential source of variation could possibly result from RuBisCO fractionation^4^ (− 27‰), which may select light isotopologues of CO_2_ for chemical reduction regardless of whether CO_2_ is enriched or depleted with respect to heavy C or O isotopes^62,63^. This “ternary effect” ^63^ is expected to be maximized when the leaf-to-air vapour mole fraction difference is greatest and the effect is thought to be most pronounced on factors derived by the difference, most notably mesophyll resistance to CO_2_ assimilation^63^. In this scenario, light isotopes of both C and O may be selected simultaneously which could theoretically contribute to correlations between δ^13^C and δ^18^O in cellulose. However, the potential net fractionation effect of this process should be much smaller than the large effect of VPD on evaporative enrichment. Indeed, a fractionation of − 4.4‰ is produced by stomatal resistance to diffusion of CO_2_ from the air into leaves^4^ (Fig. 1) but positive covariance of δ^13^C and δ^18^O does not require stomates because it is observed in pedogenic carbonate of paleosols before the evolution of stomates^62^. Since we do not consider mesophyll conductance in the model for VPD (Fig. 1) the ternary effect cannot be inferred from our data. We suggest that those potential mechanisms should be investigated experimentally in future studies to characterize their influence on δ^13^C:δ^18^O slopes in plants and soils.

Finally, it is important to note that the use of *S* as a proxy for VPD may not be suitable for application to all fossil cellulose or pedogenic carbonate samples, and several warnings are in order for application of the *S*-to-VPD transfer functions proposed here. For example, the use of these transfer functions should be limited to datasets of suitably preserved fossil specimens of known genera that show significant correlation between δ^13^C and δ^18^O values. Additionally, different soil types have inherently different abilities to hold water and nutrients, which modulates the effect of VPD on cellulose δ^13^C and δ^18^O fractionations of many dominant tree species^64^, as well as carbonate production ^33^. Thus, our results should be understood as site-specific VPD records for particularly well-studied soil types and associated plant species of interest. Furthermore, preservation bias for cellulose must also be considered. Cellulose in arid and drought-prone climates show a high positive slope between δ^13^C and δ^18^O, but in humid climates and/or waterlogged sites, *S* is generally lower and less significant (Fig. 4, Table S2). These sites and other low-VPD (< ~ 0.6 kPa) sites are among the most favorable locations to preserve cellulose because the preservation of cellulose requires exceptional taphonomic conditions that suppress decay^65^. This almost always requires rapid burial in an aqueous medium, and therefore the resulting mummified or coalified wood is likely to occur in low-VPD settings where δ^13^C and δ^18^O ratios may be decoupled^66^. Such low VPD sites include Histosol paleosols, like the Eocene *Metasequoia* wood sites used here, but *Metasequoia* stumps were emergent from the Histosol and so fully aerated (and subject to variations in atmosphere moisture), rather than completely submerged during growth^67^. Additionally, trees like *Metasequoia* cannot form woody coals unless their roots are aerated as well as their leaves^68^. Positive covariance of δ^13^C and δ^18^O in the *Metasequoia* sample implies that both roots and leaves were coupled to the atmosphere and thereby suitable for paleo-VPD estimation.

## Conclusion

A compilation of previously published data reveals positive correlations between δ^13^C and δ^18^O in response to VPD which is recorded in modern and fossil cellulose and carbonate samples. The most likely mechanisms that contribute to the correlation of δ^13^C and δ^18^O under varying VPD in plants are changes to stomatal conductance and evaporative enrichment of leaf and soil water. A third possible contribution is from leaf-level RuBisCO selection of light isotopologues of CO_2_ when the isotopic composition of the ambient air is significantly different from inside the leaf, although that effect appears to be small and unlikely to vary with VPD. Together, our results suggest that the slope of δ^13^C and δ^18^O regressions in modern cellulose and pedogenic carbonate is directly related to VPD, and thus δ^13^C:δ^18^O slope may be used to infer paleo-VPD conditions at spatiotemporal scales that go beyond those of tree-ring studies. This hypothesis is supported by a comparison of our *S*-to-VPD transfer functions applied to two fossil sites for which climate reconstructions have been previously reported. Our findings highlight the interconnectivity of the soil–plant–atmosphere system in response to atmospheric water deficit and could pave the way for the use of well-preserved fossil wood and pedogenic carbonate to estimate VPD during past climates and to improve Earth system models and their predictions of future climate.

## Methods

We compiled estimates of typical stable isotope values and fractionation steps for modern C_3_ plant cellulose^20,21^ and modern soil carbonate^22^ (Fig. 1), describing how VPD influences each isotope system. This diagram (Fig. 1) displays rough approximations for isotopic values which are expected to vary significantly with differences in geographic location, environmental conditions and concentration of atmospheric CO_2._

We use modern climate records (Table S5) along with previously published isotopic data for modern cellulose and pedogenic carbonate samples to calibrate the model used to estimate VPD with fossil samples (Fig. 4). We compiled modern (1950-present) stable isotope (δ^13^C, δ^18^O) and climate data from previously published isotopic studies of plant cellulose (n = 23) and pedogenic carbonate (n = 31) from around the world (Supplementary data). Cellulose was chosen in this study because cellulose is commonly preserved in the fossil record^39,43^, and because cellulose reflects overall trends in bulk soil organic matter variation across ecosystems^64^. Pedogenic carbonate was chosen because it is also widely observed and analyzed in the fossil record of soils^28,29,34^.

We then selected a subset of cellulose (n = 8) and carbonate (n = 13) stable C and O isotope datasets (Tables S3 and S4) that met our inclusion criteria. The criteria used for data inclusion build on previous findings that show coupling of C and O isotope excursions under drought at the molecular^8,16,18^ and ecosystem^48^ levels, for which fractionation steps have been mechanistically described (Figs. 1 and 2). For cellulose these criteria include α-cellulose from single trees (needle-bearing taxa only) collected from 1950—present that had n ≥ 8 and a significant (*P* < 0.05) positive correlation between δ^13^C and δ^18^O and met all assumptions for simple linear regression (Lack of fit test; mean of residuals is equal to 0; distributions of residuals obey normal distribution; equal variance of residuals, and low / no autocorrelation of residuals). Datasets that passed all criteria were included in the transfer function dataset (Fig. 4, Table S5). For carbonate we included only modern (Holocene) nodular pedogenic carbonate samples from a single soil profile with n ≥ 8 and a positive significant correlation and met all assumptions for simple linear regression. Both included and excluded datasets are included as supplementary material. Stable isotope values are reported or recalibrated to Vienna Pee Dee Belemnite, VPDB, for both δ^13^C and δ^18^O. The elevation, plant community, species, source water δ^18^O values and correlation coefficient of the δ^13^C:δ^18^O relationship were reported from each study (Table S5).

We use modern meteorological data (mean annual temperature [MAT], mean annual precipitation [MAP], annual relative humidity [RH], annual average vapour pressure deficit [VPD_annual_,], MAT_high_ and MAT_low_) as provided by the original authors or gathered from the closest weather station to each location (Table S5). VPD is reported in kilopascals (kPa). Modeled values of monthly maximum and minimum VPD for US locations are reported from the PRISM dataset (PRISM Climate Group, Oregon State University) and are also listed in supplementary data. We use average annual VPD for the transfer functions because the use of 50 year annual averages avoids consideration of short-term variations of source water δ^18^O^17^, and because it allowed for parity in VPD estimates across international sites. Annual average VPD, when not author- provided, was calculated using annual average annual relative humidity (RH), MAT, and saturation vapour pressure (SVP, varies as a function of MAT) and displayed in kPa using the following formula^1,2^

3 $$VPD = ((100 - RH)/100)*SVP)$$

A partial-least squares regression was performed on each modern dataset, and the slope of the δ^13^C:δ^18^O relationship (*S* as a fraction) was computed for both cellulose and pedogenic carbonate datasets that met inclusion criteria and model assumptions for ordinary least-squares regression.

Using our compiled modern cellulose dataset, we plotted δ^18^O:δ^13^C slope and annual average VPD on the modelled slopes versus VPD calibrated for *P. radiata* reported by Barbour et al.^8^ (Fig. 2e). For ease of comparison with the model^8^, we plotted our data to show δ^18^O:δ^13^C slopes (*S*^−1^), whereas δ^13^C:δ^18^O slopes (*S*) are used as previously suggested for paleo-VPD estimates. We then fit least-squares regressions to the scenarios proposed by Barbour et al.^8^ to get slopes of 0.11 when g_s_ alone varies, 0.17 when both g_s_ and V_cmax_ vary, and 1.34 when *g*_s_ and *V*_cmax_ vary while *g*_s_ varies over a limited range (Table S6).

Since VPD predictions using fossil samples assumes large uncertainties in both x and y variables, we used orthogonal least-squares regression to correlate the slope of the δ^13^C:δ^18^O relationship with the annual atmospheric vapour pressure deficit where the cellulose or pedogenic carbonate formed (Fig. 4). We accounted for uncertainty in VPD predictions by summing errors in quadrature with Gaussian error propagation (Table S5). These errors included A) the standard error of each modern data point’s δ^13^C:δ^18^O slope when slope was calculated from raw data; B) the standard error of modern VPD measurements when calculated from average climate statistics (± 0.13 kPa)^41,42^; and C) the standard error of the transfer functions. We compared the δ^13^C:δ^18^O relationship in modern and fossil samples (Fig. 5) by plotting several of the previously published isotopic datasets we included. We included the *Metasequoia* dataset because A) there was a positive correlation between δ^13^C and δ^18^O; B) it met all the inclusion criteria (Table S4); and C) it was the only dataset that provided an independent estimate for both RH and MAT for comparison to the VPD estimate presented here. Modern and fossil cellulose δ^18^O were normalized to the VPDB scale (Table S7) for comparisons with modern and fossil pedogenic carbonate δ^18^O.

## Supplementary Information

Supplementary Information.

Supplementary Information.
