# Supplementary material for: A record of vapour pressure deficit preserved in wood and soil across biomes
Source: Sci Rep. 2021 Jan 12;11:662. doi: 10.1038/s41598-020-80006-9 (PMC7804288; doi:10.1038/s41598-020-80006-9)
Supplement: Supplementary file 1 — Supplementary Information. [file 41598_2020_80006_MOESM1_ESM.pdf]

A record of vapour pressure deficit preserved in wood and soil across biomes

Adrian Broz[1], Gregory J. Retallack[1], Toby M. Maxwell[2], Lucas C.R. Silva[2]

[1] Corresponding author, Department of Earth Sciences, University of Oregon, Eugene, 97403 USA

[2]Department of Geography, University of Oregon, Eugene, 97403 USA

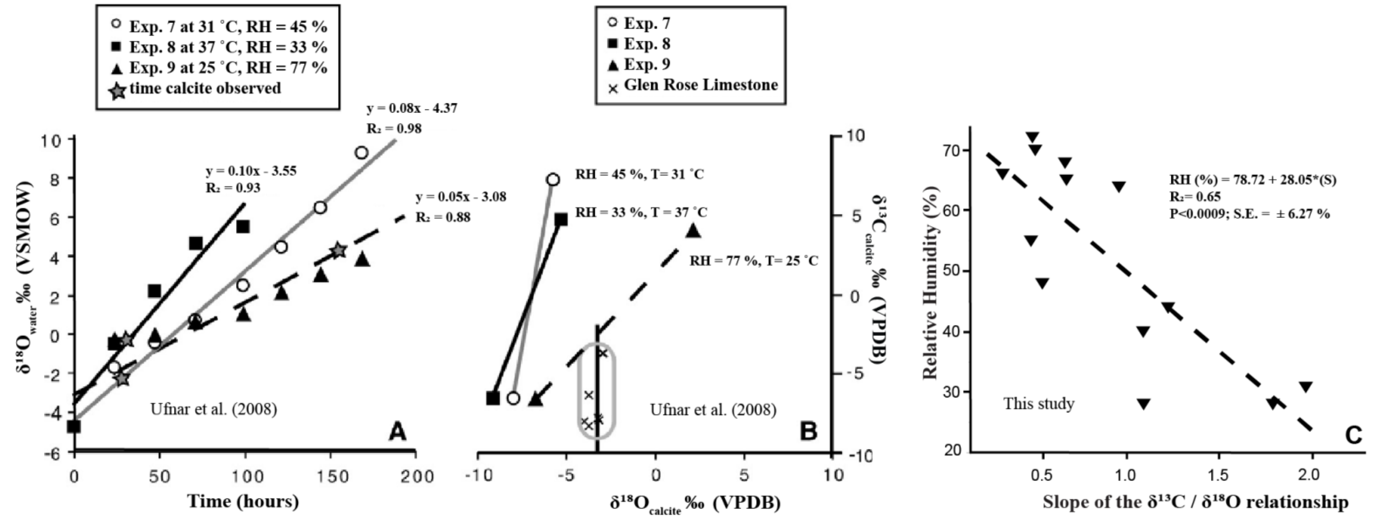

Figure S1. The slope of the linear correlation of δ^{13}C and δ^{18}O varies with relative humidity and temperature in calcite precipitated under pedogenic-like conditions in laboratory settings[35], and observations of modern pedogenic carbonate δ^{13}C and δ^{18}O slope versus annual relative humidity from this study. A) Laboratory observations of the evolution of $\delta^{18}O_{water}$ as a function of evaporative enrichment. Stars indicate when calcite crystals first appeared from evaporating fluids. B) Positive linear correlation in calcite precipitated during three experiments under vadose zone-like conditions of elevated pCO_2. Slope steepness generally increases with increasing temperature and decreasing RH. Predicted meteoric calcite values that would form in equilibrium with a $\delta^{18}O_{water}$ value of -4.8‰ at 31 °C, 37 °C, and 25 °C were calculated, then used with evaporatively produced calcites to produce positive linear correlation trends (regression lines). Crosses represent δ^{13}C and δ^{18}O of original samples of Glen Rose Formation limestone from which calcite solutions were prepared (Adapted from [35]). C) Data from this study showing a significant negative correlation between annual RH and slope in modern (Holocene) pedogenic carbonate precipitated in natural settings
